# An inventory of yeast proteins associated with nucleolar and ribosomal components

**DOI:** 10.1186/gb-2006-7-10-r98

**Published:** 2006-10-26

**Authors:** Eike Staub, Sebastian Mackowiak, Martin Vingron

**Affiliations:** 1 Department of Computational Molecular Biology, Max Planck Institute for Molecular Genetics, Berlin, Germany

## Abstract

**Background:**

Although baker's yeast is a primary model organism for research on eukaryotic ribosome assembly and nucleoli, the list of its proteins that are functionally associated with nucleoli or ribosomes is still incomplete. We trained a naïve Bayesian classifier to predict novel proteins that are associated with yeast nucleoli or ribosomes based on parts lists of nucleoli in model organisms and large-scale protein interaction data sets. Phylogenetic profiling and gene expression analysis were carried out to shed light on evolutionary and regulatory aspects of nucleoli and ribosome assembly.

**Results:**

We predict that, in addition to 439 known proteins, a further 62 yeast proteins are associated with components of the nucleolus or the ribosome. The complete set comprises a large core of archaeal-type proteins, several bacterial-type proteins, but mostly eukaryote-specific inventions. Expression of nucleolar and ribosomal genes tends to be strongly co-regulated compared to other yeast genes.

**Conclusion:**

The number of proteins associated with nucleolar or ribosomal components in yeast is at least 14% higher than known before. The nucleolus probably evolved from an archaeal-type ribosome maturation machinery by recruitment of several bacterial-type and mostly eukaryote-specific factors. Not only expression of ribosomal protein genes, but also expression of genes encoding the 90S processosome, are strongly co-regulated and both regulatory programs are distinct from each other.

## Abstract 2

Phylogenetic profiling and gene expression analysis of yeast proteins suggests that the nucleolus probably evolved from an archaeal-type ribosome maturation machinery by recruitment of several bacterial-type and mostly eukaryote-specific factors

## Background

In prokaryotes, heat and distinct ionic conditions are sufficient to assemble a ribosome from its building blocks *in vitro *[1]. In comparison, the biosynthesis of eukaryotic ribosomes is a complicated procedure. Eukaryotic ribosomes are made in the nucleolus, the ribosome factory of a eukaroytic cell. The nucleolus is a dense compartment in the nucleus of eukaryotes where freshly transcribed ribosomal RNA (rRNA) and ribosomal proteins imported from the cytosol meet complex machinery for ribosome maturation and assembly. Ribosomal subunits leave the nucleolus in a state in which the majority of their building blocks are already incorporated [2,3].

Several lines of evidence suggest that ribosome biosynthesis is not the sole function of nucleoli. They have been linked to cell growth control, sequestering of regulatory molecules (for example, of the cell cycle), modification of small RNAs, mitotic spindle positioning, assembly of non-ribosomal ribonucleoprotein (RNP) particles, nuclear export, and DNA repair [2,4-6]. The wide range of different functions linked to the nucleolus is not surprising when considering the prominent position of ribosome biosynthesis with respect to cellular economy [7]. It seems as if the regulation of a broad range of cellular mechanisms related to cell growth and division is linked to the ribosome biosynthesis machinery through nucleoli. The full range of molecules involved in this cross-talk is only beginning to emerge. Large scale proteomic analyses of nucleolar constituents [8,9] and a survey of the human nucleolar protein network [10] have recently provided a first global picture of the functional network of human nucleoli.

The baker's yeast *Saccharomyces cerevisiae *is a favorite eukaryotic model organism for ribosome-related research. However, knowledge about the set of proteins associated with ribosomes or their nucleolar precursors in yeast is fragmentary. There are currently 439 yeast proteins annotated as ribosomal, ribosome-associated, or nucleolar. Many have been identified in genome-scale protein localization studies [11,12] as well as studies of narrower focus [13-18]. Such experiments usually represent only snapshots of cells in particular states. Furthermore, native protein localization might have been altered when proteins are expressed with fusion tags or as yeast two-hybrid baits or preys. Therefore, it is likely that many additional nucleolar or ribosome-associated proteins are still undiscovered. In support of this hypothesis, studies on the proteomes of human and mouse-ear cress nucleoli [8,9,19,20] identified hundreds of proteins that were unknown before or have not yet been linked to the nucleolus. The lists of nucleolar proteins from these distantly related eukaryotes were only partially overlapping. Moreover, Andersen and colleagues [9,21] found that a large proportion of human nucleolar proteins localize to the nucleolus only transiently, which might also have rendered their discovery in yeast more difficult.

In this study, we aim to extend the fragmentary knowledge about the protein parts list of yeast nucleoli. We present a computational approach to predict novel nucleolar or ribosome biosynthesis proteins of yeast using data from orthologous nucleolar proteins and data sets on pairwise protein interactions or protein complexes. Using a naïve Bayesian classifier we predict novel proteins associated with nucleolar or ribosomal components at high estimated sensitivity and specificity. We study the evolution of these proteins using phylogenetic profiles across 84 prokaryotic and eukaryotic organisms, thereby complementing and extending earlier computational studies on the function and evolution of the nucleolus [21,22]. Finally, we investigate expression patterns of nucleolar and ribosome-associated genes to characterize the substructure of the nucleolar expression program.

## Results and discussion

### Prologue

This section is divided into three parts. In the first section, we describe a comprehensive list of yeast proteins that we predict to be associated with nucleolar or ribosomal components. Note that in the following paragraphs such proteins will be termed nucleolar or ribosomal component-associated (NRCA) proteins. NRCA proteins do not necessarily have to be associated with the ribosome or to be localized in nucleoli during their whole life cycle. Instead, it is possible that a predicted NRCA protein localizes to the nucleolus only temporarily or binds to nucleolar components outside the nucleolus. All proteins that associate with ribosomal and nucleolar components are the targets of our predictions. In this way we would like to capture all proteins that have the potential to exert important functions on nucleolar and ribosomal biology. In the second part of the study, the identified proteins are subjected to phylogenetic profiling, thereby providing insights into the evolution of the nucleolus and ribosome assembly. Finally, we characterize the gene expression program for NRCA proteins by comparison of expression patterns of diverse functionally or evolutionarily related sets of genes.

### Prediction of novel nucleolar and ribosome-associated proteins

**Figure 1:**
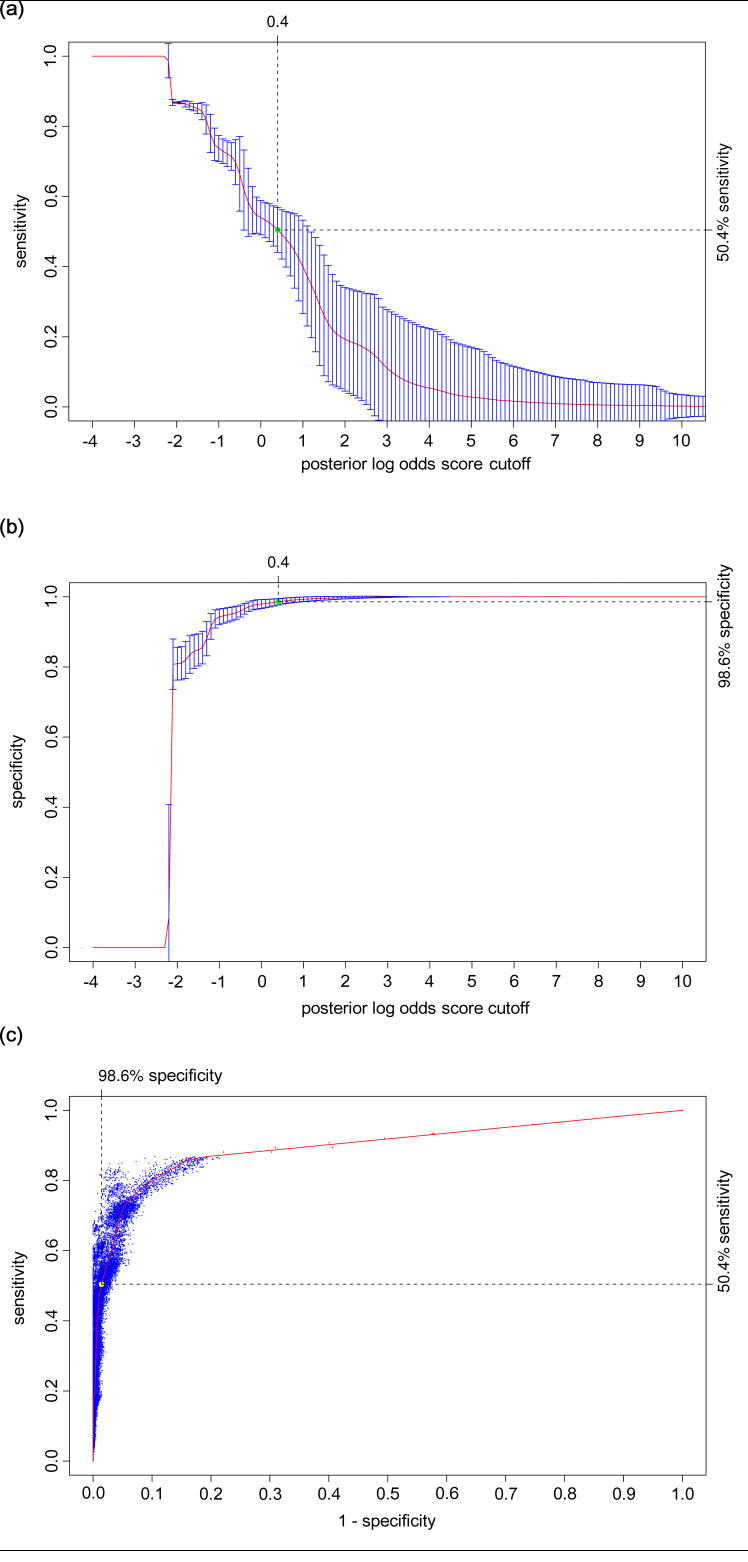
**Estimation of prediction accuracy**. The accuracy of predictions was estimated from 1,000 runs of 10-fold cross-validations using 1,000 alternative training sets (see Materials and methods). The threshold/working point used for the final predictions of new nucleolar proteins is marked in each plot. **(a) **The sensitivity (*SE *= *TP*/(*TP *+ *FN*)) of our classifier is plotted over different thresholds of classifier scores (log posterior odds ratios) applied to each cross-validation run. The logarithmic posterior odds ratios indicate how likely it is under the naïve Bayesian model that a protein is an NRCA protein (positive scores) versus that it is not an NRCA protein (negative scores). A single point on the line and its error bar stems from calculations of the average sensitivity and its standard deviation obtained from 1,000 cross-validation runs using a distinct classification score threshold. Confidence intervals are ± 2-fold standard deviation intervals around the mean. Note that at the threshold that was finally used for prediction (0.4) we expect to reach a sensitivity of 50.4%. This means that we have probably still missed as many NRCA proteins as we have predicted (62). **(b) **The specificity (*SP *= *TN*/(*TN *+ *FP*)) of our classifier is plotted over different thresholds of classifier thresholds (log posterior odds ratios) that were applied on results of each of 1,000 cross-validation runs. Confidence intervals are ± 2-fold standard deviation intervals around the mean. Note that at the finally used threshold of 0.4 the specificity reaches 0.986, meaning that we expect only 1.4% of false positives among our predictions. **(c) **The ROC curve of our classifier is plotted as sensitivity versus (1-specificity). Each individual data point reflects predictions at a single cross-validation run when a single prediction threshold is applied. The central line is based on averaged SE/SP values for each threshold applied. The ROC curve gives an impression of the quality of a classifier. It is a general indicator of classification performance. The bigger the AUC, the better the classifier. We obtained an AUC value of 0.98, which generally indicates a classification of high quality. The ROC curve was also the basis for the selection of our final classifier threshold, as it illustrates the trade-off between sensitivity and specificity. We chose to be very conservative (high specificity) for the sake of missing true NRCA proteins (lower sensitivity).

A prerequisite for comprehensive functional and evolutionary characterization of the nucleolus and the ribosomal machinery is a complete parts list of its proteins. We applied naïve Bayesian classification to extend the known list of 439 proteins associated with nucleolar and ribosomal components in yeast towards a complete inventory of such proteins. Before prediction of new factors, we performed an extensive cross-validation of our naïve Bayesian classifier to judge whether we are able to predict NRCA proteins with considerable accuracy (Figure 1). To this end, we built 1,000 training sets, performed a cross-validation and obtained 1,000 receiver operating characteristic (ROC) curves. The average area under the ROC curve (AUC) was approximately 0.98, which generally indicates a classifier of high performance. Based on cross-validation and ROC analysis on the training sets, we chose a conservative threshold of *log*(*O*_*post*_) > 4 for the prediction of new NRCA proteins. During cross validation we predicted nucleolar proteins at a sensitivity of 50.4% and a specificity of 98.6% using this threshold, indicating that our predictions are very conservative.

**Figure 2:**
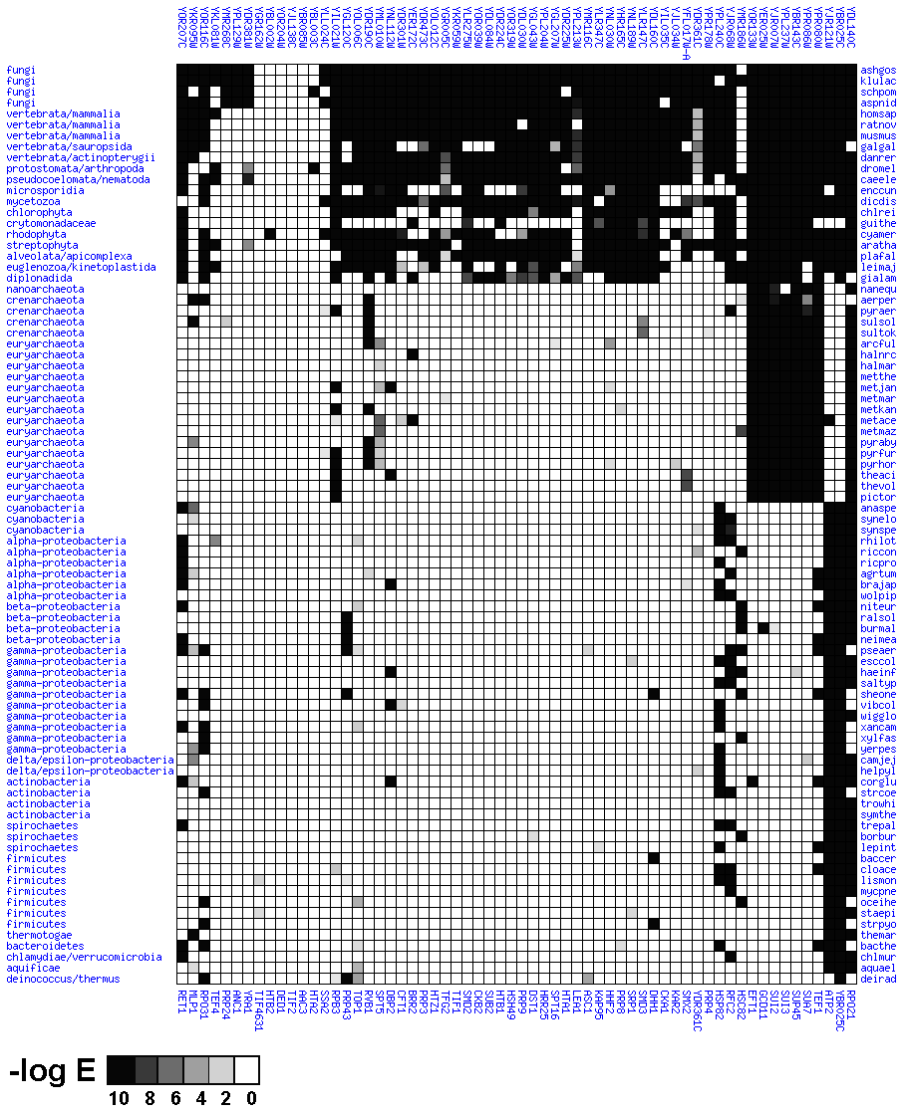
**Phylogenetic profiling of novel nucleolar/ribosome-associated proteins**. Phylogenetic profiles of 62 previously unrecovered nucleolar/ribosome-associated proteins of yeast across 84 organisms. The profiles were generated using the best reciprocal hit method with yeast as a reference organism (see Materials and methods). Abbreviations given on the top of the plot represent organism names (first three letters for genus and first three letters of species names; see Materials and methods for a translation of abbreviations into organism names). Further taxonomic annotation is given on the bottom of the plot. Yeast open reading frame identifiers are given on the left side, and gene names and descriptions are given on the right side of the plot. The significance of sequence similarity is visualized by different shades of gray that reflect the logarithmic expectation (E) value from reciprocal BLAST searches (shown at the bottom of the figure). Here, the E values of BLAST searches using target proteome sequences as queries versus the yeast proteome reference database are shown. The genes are ordered according to hierarchical clustering (see Materials and methods).

Out of 6,281 proteins that were not annotated as NRCA proteins before, we predicted a further 62 to be linked to nucleolus/ribosome biology (Table 1, Figure 2). The experimental evidence underlying our predictions can be encoded in a 7-bit binary data string. All data strings that occurred in our analysis are summarized in Table 2 along with the prediction results obtained for them. When sensitivity/specificity estimates of the cross-validation runs hold, we estimate that there is approximately 1 false positive prediction among the 62 proteins and that we missed about another 62 proteins by our approach. We conclude that the complete inventory of nucleolar and ribosome-associated proteins in yeast comprises 439 previously known proteins, 62 predicted in this analysis, and about another 62 proteins that remain to be discovered. Thus, we hypothesize that, in total, approximately 560 genes (more than 8% of the total gene content) encode proteins related to nucleolar or ribosomal biology in yeast.

The majority of newly predicted NRCA proteins belong to four functional classes. The first class consists of proteins that were known as regulators of translation before: the translation initiation factors TIF1, SUI3, SUI2, TIF2, GCD1, TIF4631, the translation elongation factors TEF1, TEF4, EFT1, SPT5, the translational release factor SUP45, and the translocon component KAR2. We identified these proteins not only because of their physical interactions with other translation factors or ribosome components, but also because each factor has orthologs in human and/or mouse-ear cress that have been detected in nucleoli. Although the ribosomal association of these factors was known before, their appearance in the nucleolus is surprising. It lends further support to the hypothesis that ribosomal subunits in the nucleus already have translational competence [23-25]. Alternatively, the nucleolar translation factors could support the assembly or quality control of ribosomes, for example, by ensuring through their physical presence that their future binding sites are assembled and modified correctly.

The second class comprises factors that are linked to transcription. Whereas RNA polymerase I is the natural polymerase for the transcription of rRNA genes in the nucleolus, we additionally predicted the nucleolar association of the RNA polymerase II factors SUA7, RPO21, DST1, TFG2, RPB3, TIF4631, and TAF14, and the RNA polymerase III factors RPO31 and RET1. Several of these factors (RPO21, TIF4631, TAF14, RPB3, RPO31, RET1) have not been identified in nucleolar preparations, but were linked to other nucleolar proteins by shared participation in protein complexes and/or interactions in independent experiments. Therefore, it is possible that they associate with nucleolar/ribosomal proteins only outside the nucleolus. The remaining factors were all identified in at least one nucleolar purification experiment, suggesting that they could play yet undiscovered roles as regulators of ribosomal gene expression by RNA polymerase I.

As a third group, we predicted several components of the splicing apparatus to occur also in the nucleolus [26,27]. Among these are components of the major spliceosomal subcomplexes, namely the U1 small nuclear (sn)RNP protein SMD2, the U4/U6 snRNP factors PRP3 and PRP4, the U2A snRNP protein LEA1, the U2 components PRP9 and HSH49, the U5 snRNP protein PRP8, and the Sm core proteins SMX2 and SMD3. Furthermore, we predict the nucleolar localization of the exon junction complex component SUB2 and the spliceosome disassembly protein PRP43. U3 snRNP proteins are already known to contribute to early steps in ribosome assembly and are components of the 90S processosome. We propose that the identified spliceosomal proteins have as yet unknown functions in the assembly of ribosomes and/or other nucleolar RNPs.

The fourth class is linked to the regulation of genomic DNA structure and chromatin. The nucleolar association of several nucleosome components like histone H2A.2 (HTA2), H4 (HHF2), H2B.2 (HTB2), H2B (HFB1), and an H2A variant of the F/Z family (HTZ1) is not surprising as genomic DNA is an integral part of nucleoli that are formed by fusion of so-called nucleolar organizer regions (NORs), stretches of genomic DNA carrying rRNA genes. DNA topoisomerase I (TOP1) could be required to relax tension in DNA structure in NORs, either during replication or transcription. SPT16 is an essential general chromatin assembly factor that is known to assist in RNA polymerase II transcription. Rvb1p (RVB1) is also essential for yeast viability and known as a component of chromatin remodeling complexes. Our results suggest that both proteins are involved in remodeling the chromatin of NORs.

Putative biochemical functions of several further predicted nucleolar proteins are in accordance with a role in nucleolus or ribosome maturation. The gene *DHH1 *encodes an RNA helicase of the DEAD box family that was not found in nucleoli of ear cress or human, but interacted with known nucleolar proteins in four independent data sets (Table 1). Another DEAD box RNA helicase encoded by *DBP2 *was found in nucleoli and in nucleolar complexes. In combination with their putative biochemical function, this is strong evidence that both RNA helicases play a role in nucleolar RNP assembly. The *BCP1 *gene is largely of unknown function, but its deletion is lethal in yeast. It has been linked to nuclear transport and maturation of ribosomes through interactions with a ribosomal lysine methyltransferase (RKM1), to a RAN-binding protein (KAP123), to a ribosomal protein (RPL23A) and to its essentiality for nuclear export of the Mss4p protein. Although little is known about the cellular function of the heat shock proteins HSP82 and SSA2, their occurrence in nucleoli is not surprising because protein folding is a fundamental process during RNP assembly. Similarly, it seems reasonable to assume a ribosomal function for the karyopherins alpha and beta (KAP95, SRP1). The Uso1p-related myosin-like protein (MLP1) is linked to the interior side of the nuclear envelope and nuclear pore. It is proposed to act in the nuclear retention of unspliced messengers. Its identification in nucleolar preparations suggests that it fulfills a similar role in the control of RNA or RNP processing in the nucleolus.

Furthermore, there were several surprising predictions of novel nucleolar proteins. Two subunits (CKA1 and CKB2) of yeast casein kinase 2 (CK2) were predicted to be nucleolar. CK2 is known as a pleiotropic regulator of the cell cycle and has recently been linked to the regulation of chromatin [28]. Therefore, we hypothesize that CK2 regulates chromatin accessibility in nucleolar organizer regions. Casein kinase 1 is known for its function in intracellular vesicle transport and secretion [29]. A nucleolar role of casein kinase 1 (HHR) was not known during preparation of this manuscript, but was published during the revision stage (see Note added in proof). An F1 beta subunit component of the F1F0-ATPase complex (ATP2) has been detected in nucleolus purifications of both ear cress and human. This strongly suggests a dual function for this protein in respiration and the nucleolus. The nucleolar localization of a mitochondrial ADP/ATP carrier protein (AAC3) was also detected in both model organisms and is supported by protein interactions to nucleolar proteins.

We note that, in total, only 11 of 62 proteins have been identified solely on the basis of protein interactions; the remaining 51 proteins have nucleolar orthologs in model species. We expect that the latter perform yet undiscovered functions in the nucleolus, although they have been linked to extra-nucleolar or even cytosolic processes like splicing, nuclear ribosome import/export, or translation before. The former are candidates for yeast-specific nucleolar localization or for extra-nucleolar ribosome maturation. Further functional characterization is hardly possible using only presently available data and would, therefore, require additional experiments.

### Note added in proof: validation of our predictions in the current literature

During revision of this manuscript we became aware of several old and new articles that add experimental evidence to some predictions of nucleolar or ribosome-associated proteins made in this manuscript. We were not of aware of the ribosomal or nucleolar roles of these proteins before, because such annotations were missing in the *Saccharomyces *Genome Database (SGD) database at the time of analysis. In the following we shortly describe these findings of others. Lebaron *et al*. [30] and Leeds *et al*. [31] found that the Prp43 protein, a putative DEAH helicase, is a component of multiple pre-ribosomal particles and localizes to the nucleolus. We predicted a nucleolar role of Prp43 via evidence from nucleolar preparations in model organisms and from protein-protein interactions. Schafer *et al*. [32] have shown recently that the protein kinase HRR25 (casein kinase I) binds pre-40S particles, phosphorylates Rps3 and the maturation factor Enp1, and is required for maturation of the 40S subunit *in vivo*. We predicted a ribosomal/nucleolar role for HRR25 based on the occurrence of the human HRR25 ortholog in nucleolar preparations and on the co-occurrence of HRR25 with other nucleolar proteins in affinity-purified protein complexes (Table 1). In 2001, Bond *et al*. [33] had already shown that DBP2 is not only involved in nonsense-mediated mRNA decay, but is also a ribosome biogenesis factor as DBP2 mutant cells are deficient in free 60S subunits and 25S rRNA is significantly reduced. This link has apparently escaped the attention of SGD database curators for years. We rediscovered the link of DBP2 with ribosomal biology through a prediction based on nucleolar localization of the human DBP2 ortholog and through interactions with nucleolar proteins in protein complex data of two independent studies (see table 1). In 2000, Edwards *et al*. [34] found that yeast topoisomerase TOP1 localizes to the nucleolus dependent on its interaction with nucleolin. We rediscovered this link because of the co-occurrence of yeast TOP1 in protein interactions and complexes with nucleolar components and the nucleolar localization of human TOP1. These four cases are independent experimental validations of our predictions.

### Phylogenetic profiling of nucleolar and ribosome-associated proteins

**Figure 3:**
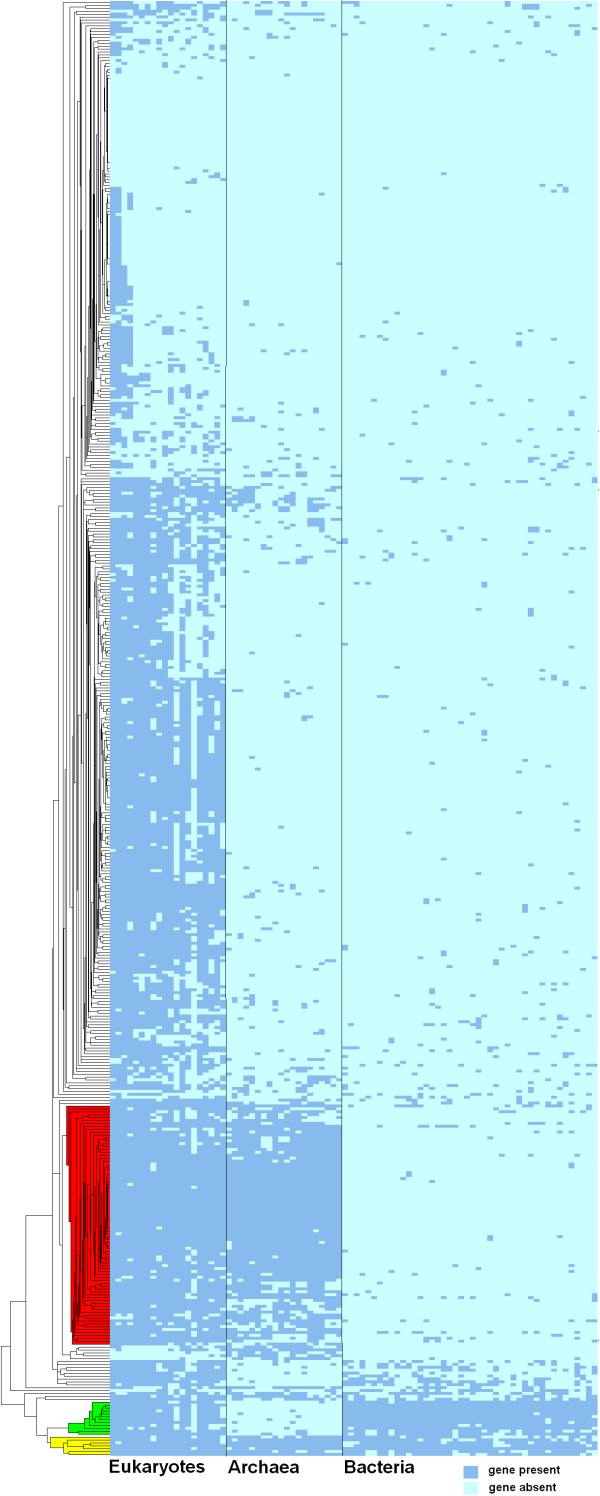
**Hierarchical clustering of phylogenetic profiles of nucleolar proteins**. Phylogenetic profiles of all 501 nucleolar or ribosome-associated proteins. Organisms vary along the horizontal axis, proteins along the vertical axis. Presence of a gene is indicated by dark blue, absence by light blue. Organisms from the three domains of life are separated by black bars. The dendrogram resulting from protein-wise hierarchical clustering is given on the left. Several evolutionarily meaningful clusters emerged, which are colored in the dendrogram: red, proteins of archaeal origin; yellow, ubiquitous proteins; green, proteins of (eu-)bacterial origin. Note that the eukaryote-only genes constitute the largest group, followed by the archaea/eukaryote group. There is a considerable number of genes with orthologs only in bacteria and eukaryota, but not in archaea.

**Figure 4:**
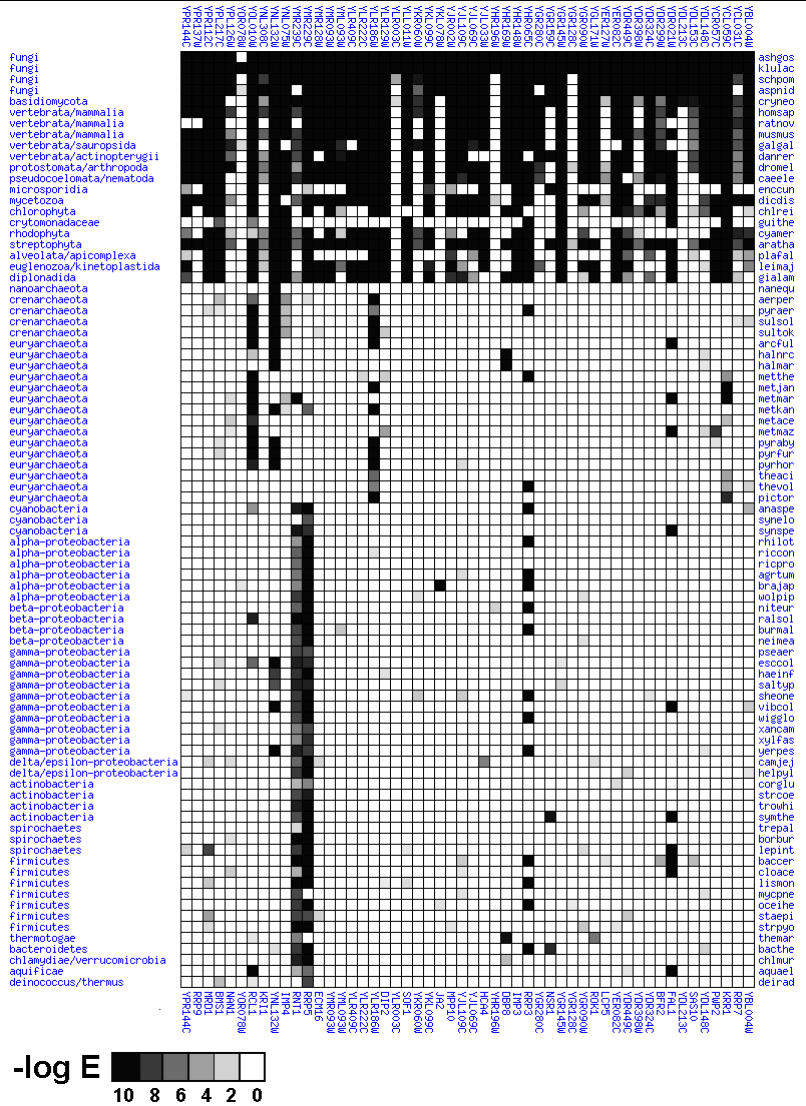
**Phylogenetic profiling of the 90S processosome**. Phylogenetic profiles of known yeast 90S processosome proteins across 84 organisms. Abbreviations given on the top of the plot represent organism names (first three letters for genus and first three letters of species names; see Materials and methods for a translation of abbreviations into organism names). Further taxonomic annotation is given on the bottom of the plot. Yeast open reading frame identifiers are given on the left side, and gene names and descriptions are given on the right side of the plot. The significance of sequence similarity is visualized by different shades of gray that reflect the logarithmic expectation (E) value from reciprocal BLAST searches (shown at the bottom of the figure). Here, the E values of BLAST searches using target proteome sequences as queries versus the yeast proteome reference database are shown. The genes are ordered according to hierarchical clustering (see Materials and methods). Note that there are only a few proteins with many prokaryotic orthologs when compared to Figure 3.

We established presence-absence patterns of genes across multiple organisms, so called phylogenetic profiles, for all 501 NRCA proteins (Figures 2, 3, 4) to investigate their ancestry in the three domains of life. We identified a large cluster of 83 yeast proteins by hierarchical clustering with orthologs in the majority of archaeal species under investigation, but only single orthologs in bacteria (Figure 4). Among the archaeal proteins were many maturation factors and components of the ribosome. From a biochemical viewpoint, together with a few proteins that are ubiquitous in all domains of life, these archaeal-type proteins seem to represent the functional core of the nucleolus and of ribosome maturation.

There is a considerable, but much smaller, fraction of nucleolar proteins that have orthologs in bacteria, but not in archaea (Figure 3). Among these are RRP5, which is essential for the processing of 18S and 5.8S rRNA [35], and the 3'-5' exonuclease DIS3, which is required for the processing of 5.8S rRNA and is a component of the exosome [36]. Eukaryotes have employed these bacterial-type proteins for the processing of archaeal-type ribosomes. More detailed phylogenetic studies will have to show whether these bacterial-type proteins are even of alpha-proteobacterial (that is, mitochondrial/hydrogenosomal) origin. Interestingly, several proteins of mitochondrial ribosomes seem to localize to the nucleolus (MRPS28, MRPL9, MRPL23, YML6). Unlike most other mitochondrial ribosomal proteins, YML6 is essential for yeast viability, indicating that it does not exclusively function in mitochondria. The dual nucleolar and mitochondrial localization of these proteins means that they have taken over important functions in nuclear ribosome maturation in addition to their roles in mitochondrial ribosomes. RNAase III encoded by the *RTS1 *gene is involved in the processing of U2 snRNA, highlighting also the chimeric evolutionary origin of the machinery for RNA splicing. The tRNA-isopentenyl-tranferase MOD5 is known as one of the few proteins that occur in three subcellular compartments: cytosol, mitochondria, and the nucleus [37]. Its phylogenetic profile shows that MOD5 shares a common sequence ancestor with bacteria. The finding that eukaryotes employed bacterial-type, possibly mitochondrial, proteins to supplement the archaeal-type ribosome maturation machinery is congruent with earlier observations on the level of protein domains [22].

The largest fraction of yeast NRCA proteins has multiple orthologs in eukaryotes, but none in prokaryotes. Many of these proteins can be regarded as eukaryotic inventions. This group spans the whole range of nucleolar and ribosome-related functions. Explicitly, we investigated the profiles of components of the 90S processosome, a large complex attached to freshly transcribed rRNA that performs early maturation steps before ribosomal proteins and rRNA are assembled into subunits. The 90S processosome proteins do not show strong similarity to prokaryotic proteins, although they are strongly conserved in eukaryotes (Figure 4). As ribosome assembly in eukaryotes is much more complex than in prokaryotes, the finding that the 90S processosomal machinery has no prokaryotic counterpart is not surprising. It shows that a large machinery of proteins acting in concert at an early step during ribosome maturation has been invented exclusively for the eukaryotic branch of life.

### Implications for hypotheses on the origin of eukaryotes

What do all these results mean with respect to hypotheses about the origin of eukaryotes? Although a phylogenetic profile can reveal a prokaryotic ancestry, it can not prove a prokaryotic origin of a nucleolar protein. This question has to be studied for all proteins by single phylogenetic analyses that are beyond the scope of this study. When the first genomes were available in the late 1990s, sequence comparisons led to the postulates that 'informational' proteins in eukaryotes stem from archaea and 'operational' proteins stem from bacteria and several authors have put forward hypotheses on the origin of eukaryotes based on 'genome fusion' [38-42]. Kurland *et al*. [43] have recently called these interpretations into question and argued that whole-genome sequence comparisons, many phylogenetic analyses (in which eukaryotic proteins do not branch within archaeal or bacterial orthologs), and so called eukaryote-specific cellular signature structures (CSSs) rather show that eukaryotes represent a primordial lineage and are not just an amalgamation of prokaryotic genomes. According to another recent hypothesis, eukaryotes, archaea and bacteria each evolved by independent transitions from the RNA world to the DNA world through viral transduction [44]. The latter two hypotheses postulate that eukaryotes comprise a lineage as equally old as bacteria and archaea and are, hereafter, referred to as 'primordial eukaryote' hypotheses.

According to 'genome fusion' hypotheses, the existence of nucleolar proteins of archaeal and bacterial type would mean that the nucleolus is chimeric, with building blocks acquired from both archaea and bacteria. In contrast, 'primordial eukaryote' hypotheses would either explain prokaryotic-type proteins by gene uptake (either by horizontal gene transfer, viral transfer or endosymbiosis) or by common ancestry with genes in the last universal common ancestor (LUCA) with subsequent loss in either the bacterial or archaeal lineage.

The fact that the largest fraction of nucleolar proteins lacks counterparts in prokaryotes suggests that the nucleolus is primarily a eukaryotic invention. According to 'genome fusion' hypotheses, the many eukaryote-specific nucleolar proteins would have evolved after the genome fusion that led to the first eukaryote, thus at a relatively late time point in evolution. According to the 'primordial eukaryote' view, eukaryote-specific nucleolar proteins would be as equally old as the prokaryote-type proteins and should also be witnesses of early eukaryote (and even earliest cellular) evolution.

So far, considerations based on phylogenetic profiling do not rule out either type of hypothesis. However, our study also shows that proteins of the functional core of nucleoli are not distributed evenly across the three evolutionary groups (archaeal like, bacterial like, eukaryote specific). It is the archaeal-like set of proteins in combination with the ubiquitous proteins that represent the functional core of nucleoli and ribosome maturation. This leads us to the postulate that bacterial-type and eukaryote-specific proteins were assembled around an archaeal-type functional core, and, therefore, emerged later in the ribosome maturation machinery. How does this fit into the different types of hypotheses?

The timely order of cellular transitions outlined above would fit the 'genome fusion' hypotheses in which nucleoli evolved as a compensatory mechanism to prevent dilution of ribosome assembly factors in an early eukaryotic lineage [22]. This would have been necessary to maintain the efficiency of ribosome assembly in eukaryotes. At some time point the eukaryotic lineage must have evolved towards larger cell sizes, a development made possible by more efficient catabolism via mitochondria or hydrogenosomes [22]. In this scenario, nucleoli have emerged after the mitochondrial precursor symbiont entered its host cell, probably as a result of special pressure exerted by larger cell volumes.

Under such a hypothesis of nucleolar evolution based on 'genome fusion' it is possible that eukaryotes with mitochondria (or mitochondrial/hydrogenosomal remnants) exist that have never evolved nucleoli. In contrast, eukaryotes with nucleoli and without mitochondria would not be compatible with the hypothesis. Today, the existence of a eukaryote that lacks either mitochondria or nucleoli (or remnants of them) has not been proven [45]. Recently, Xin *et al*. [46] described a typical nucleolar protein in *Giardia lamblia *and concluded that Giardia once had nucleoli. We conclude that, so far, 'genome fusion' hypotheses are compatible with current data on nucleolar evolution.

'Primordial eukaryote' hypotheses presented so far have been less specific about the timely order of events that generated eukaryotic signature structures. Also, the driving forces that led to major eukaryotic signature structures have not been proposed. Hypotheses that postulate a eukaryotic 'raptor' as the host cell that acquired mitochondria imply that a nucleolus and nucleolar structures like 90S processosomes (eukaryotic signature structures) preceded mitochondria. This means that all eukaryotes with mitochondria should also have nucleoli or nucleolar remnants. It seems as if all known eukaryotes fulfill this criterion. However, unlike for 'genome fusion' hypotheses, one might argue that eukaryotes with nuclei and nucleoli that never had mitochondria/hydrogenosomes should have survived until today. But, as many recent studies have shown, the existence of such eukaryotes has not so far been proven [45].

In summary, our phylogenetic profiles are not sufficient to rule out either 'primordial eukaryote' or 'genome fusion' hypotheses. However, each future hypothesis about eukaryotic origins would also have to explain the hallmarks of nucleolar evolution highlighted above, that is, the archaeal nature of the functional core of nucleoli to which bacterial-type additions and many eukaryote-specific proteins were recruited.

### Distinct nucleolar gene expression programs: the ribosome and the 90S processosome

**Figure 5:**
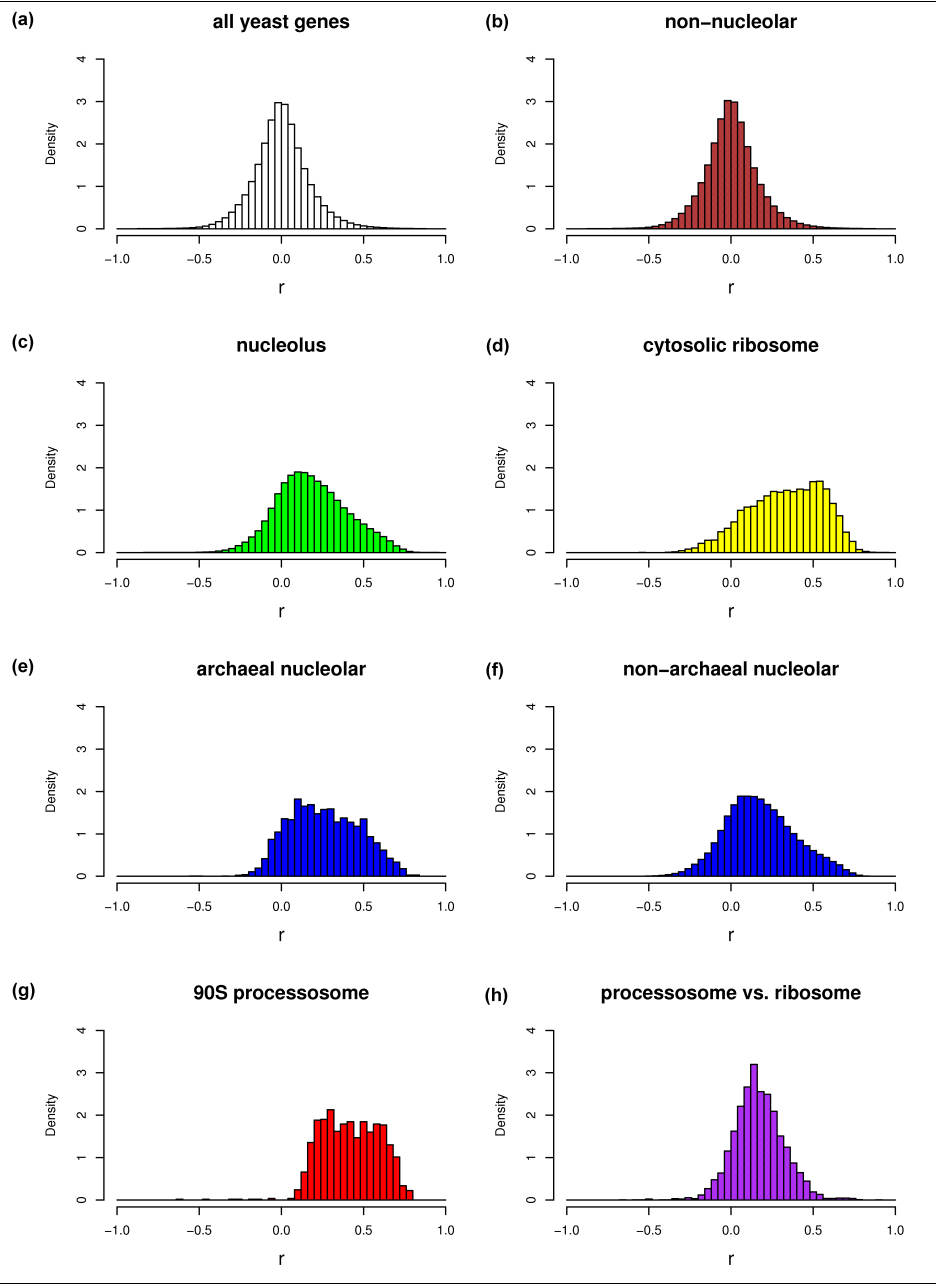
**Survey of nucleolar/ribosomal gene expression**. Histograms of sets of pairwise Pearson correlation coefficients computed from vectors of gene expression ratios for gene pairs. The distributions of Pearson correlation coefficients (each obtained from the pairwise comparison of expression profiles of two genes) gives an impression of the global similarity of expression patterns in a group of genes. Random data would give a Pearson correlation coefficient distribution centered around 0 (no correlation). The more a distribution deviates towards +1 compared to a 0-centered bell shape, the more similar a group of genes is expressed across the whole expression compendium. Gene pairs were formed within or between the functional/evolutionarily-defined groups of genes that are under investigation here. **(a) **Correlation within all yeast genes. **(b) **Correlation within genes that do not encode nucleolar proteins. **(c) **Correlation within genes for nucleolar proteins. **(d) **Correlation within genes for ribosomal or ribosome-associated proteins. **(e) **Correlation within nucleolar genes that stem from archaea. **(f) **Correlation within nucleolar genes that do not stem from archaea. **(g) **Correlation within genes that encode 90S processosome components. **(h) **Correlation between genes for ribosome proteins and 90S processosome proteins. Note that the distributions for the ribosomal protein genes and the 90S processosome strongly deviate from the rather 0-centered distribution of 'all genes-versus-all gene' comparisons. However, the distribution for gene pairs in which one partner is a 90S processosome component and the other partner is a ribosomal component deviate much less from the random shape and, thus, indicate distinct expression programs.

The expression compendium of Hughes *et al*. [47] reflects a considerable part of the global yeast expression program. We studied this data set to identify particular groups of yeast genes that are expressed similarly across the 300 experiments of this global genetic perturbation study (Figure 5).

First, we compared the correlation of expression between genes that encode nucleolar and ribosome-associated proteins with the correlation within all other yeast proteins. There is considerably higher correlation of expression patterns among NRCA protein-encoding genes, suggesting that there is a special nucleolar expression program. One might suspect that the ancient archaeal core of nucleoli, which includes many ribosomal proteins and maturation factors, constitutes a nucleolar subcomponent that exhibits an especially high degree of expression co-regulation. Therefore, we divided our set of nucleolar/ribosome-associated proteins into an archaeal set and a non-archaeal set and compared the correlation of expression within these groups. The distributions of correlation coefficients look rather similar, suggesting that evolutionary age or sequence conservation is not paralleled by tight expression co-regulation.

In contrast, the protein components of the ribosome show a marked co-regulation that is much stronger than the co-regulation observed for all nucleolar proteins. The 90S processosome is a large particle formed around unprocessed rRNA (see previous section). Surprisingly, we found that the degree of co-regulation among 90S processosomal genes is comparable with, if not higher than, that among ribosomal protein genes. We next asked whether co-regulation of ribosomal and 90S-processosomal genes is coupled, that is, whether they are under the control of the same expression program. The cross-comparison of expression vectors of genes from both particles suggests that their expression is different (Figure 5).

**Figure 6:**
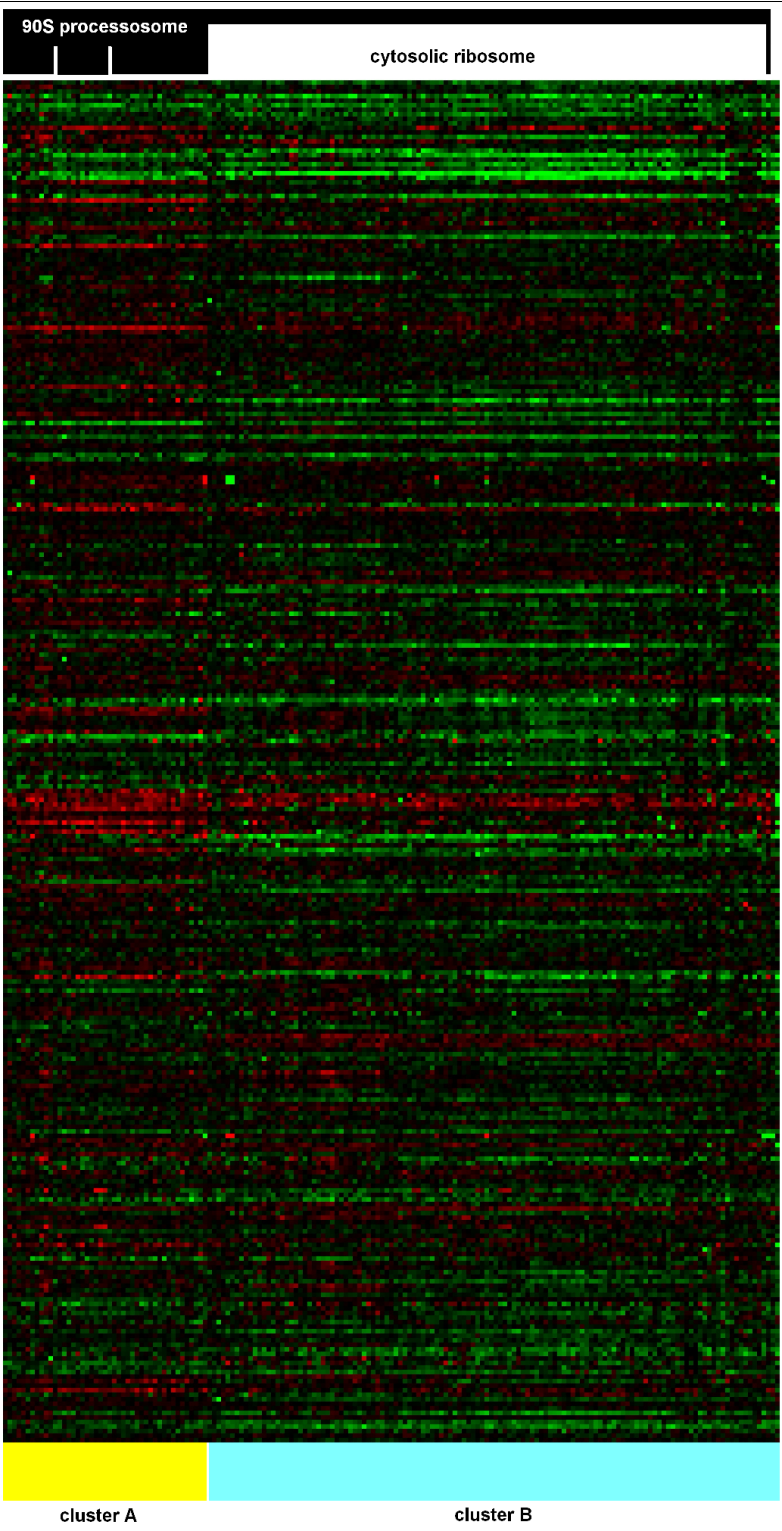
**Hierarchical clustering of gene expression patterns of ribosomal and processosomal protein genes**. The central plot shows color-coded expression ratios as supplied in the ROSETTA expression compendium [47] for genes encoding ribosomal and 90S-processosomal proteins. Genes vary along the horizontal axis, expression experiments vary along the vertical axis. Top: 90S-processosomal genes are marked in black, ribosomal protein genes are marked in white. Bottom: hierarchical clustering yields two large clusters, here marked in cyan and in yellow, that comprise approximately 80% of all ribosomal/processosomal genes (171 of 211). Only genes of these clusters are shown here. Note that only three genes are not clustered according to their membership to either the ribosome or the 90S processosome. The separation of the 90S processosomal and ribosomal protein genes by hierarchical clustering (an unsupervised approach) confirms that the ribosomal and 90S processosomal expression programs are distinct from each other (Figure 5).

We examined this difference in more detail by unsupervised clustering of expression data for a fused list of genes from both large complexes (Figure 6). Hierarchical clustering revealed that the majority of genes were distributed among two large clusters. One cluster was composed nearly entirely of ribosomal proteins, and the other cluster nearly entirely of 90S processosome proteins. We concluded that the 90S-processosomal expression program is highly co-regulated, but different from the ribosomal program. Thus, the 90S processosome proteins not only differ from their ribosomal functional associates with respect to evolution (see above), but also with respect to gene expression. We note that there is a large overlap between the sets of proteins of the 90S processosome and the so-called Ribi (ribosome biosynthesis) regulon [48-50]. Of 52 proteins of the 90S processosome, 46 are also components of the Ribi regulon. We propose that the 90S processosomal genes constitute a functionally defined module of the Ribi regulon.

Furthermore, phylogenetic profiles suggest that most 90S processosome components are not just remnants of prokaryotic precursor proteins that could stem from an amalgamation of archaeal and bacterial contributions during the origin of eukaryotes. The eukaryote-specific conservation of many 90S processosome proteins rather suggests that the 90S processosome emerged solely during eukaryotic evolution. Thus, the 90S processosome can be regarded as an ancient eukaryote-specific functional module.

## Conclusion

Baker's yeast is the major model organism for the study of eukaryotic nucleolar processes, in particular the assembly of ribosomes. However, recent studies in other eukaryotic model organisms suggest that only a fraction of nucleolar and ribosome biogenesis proteins of *S. cerevisiae *is known today. Using large-scale data sets of nucleolar proteins in *Homo sapiens *and *Arabidopsis thaliana *and protein interactions and complexes in *S. cerevisiae*, we predicted with high confidence that 62 further proteins are associated with nucleolar or ribosomal components, thereby extending the list of nucleolar/ribosomal component-associated proteins to 501. A survey of their presence-absence patterns across 84 organisms from all domains of life confirmed a shared ancestry of the nucleolar functional core with archaea. It also revealed several additions of bacterial character, and that the majority of nucleolus- and ribosome-associated proteins in yeast are eukaryote-specific. Proteins of the 90S processosome tend to be conserved across eukaryotes, but not in prokaryotes. In summary, this suggests an exclusive emergence of many nucleolar ribosome maturation factors in the eukaryote lineage. These findings represent novel insights into transitions leading to eukaryote-specific structures and represent cornerstones that have to be addressed by future hypotheses on the origin of eukaryotes. Furthermore, the analysis of a public gene expression compendium revealed that genes encoding the 90S processosome are nearly as tightly regulated as genes encoding ribosomal proteins, but that the gene expression programs of the ribosome and 90S processosome are distinct.

## Materials and methods

### Compiling data for classification

Let each yeast protein have an associated data vector x→
 MathType@MTEF@5@5@+=feaafiart1ev1aaatCvAUfeBSjuyZL2yd9gzLbvyNv2Caerbhv2BYDwAHbqedmvETj2BSbqee0evGueE0jxyaibaiKI8=vI8tuQ8FMI8Gi=hEeeu0xXdbba9frFj0=OqFfea0dXdd9vqai=hGuQ8kuc9pgc9s8qqaq=dirpe0xb9q8qiLsFr0=vr0=vr0dc8meaabaqaciGacaGaaeqabaqadeqadaaakeaaceWG4bGbaSaaaaa@343C@_*k *_= {*x*_1_, *x*_2_, ..., *x*_*K*_} with *x*_*k *_denoting an individual experimental observation for experiment *k*. This binary data vector carries information that will be used to predict whether a single protein is nucleolar or not. The total number of different sources of evidence is K. Annotations of nucleolar localization for 439 yeast proteins were retrieved from the SGD. We used seven sources of evidence (*K *= 7), hereafter also termed data columns, to judge whether a yeast protein is likely to be nucleolar or not. By default, all observations were set to *x*_*k *_= 0.

Data columns *k *= 1, *k *= 2 contain information on whether a yeast protein has an ortholog in human/*Arabidopsis *that has been detected in purified nucleoli of these organisms by mass spectrometry. We determined orthology relationships between yeast proteins and human/*Arabidopsis *proteins using INPARANOID [51]. If a yeast protein has an ortholog in a model organism that was found in nucleoli, we set the observation in the associated data vector to *x*_*k *_= 1. For data columns *k *= 3, *k *= 4 we used the yeast two-hybrid data sets for protein-protein interactions of Uetz *et al*. [52] and Ito *et al*. [53]. Whenever a yeast protein was involved in a pairwise protein interaction with another protein that is among the 219 known nucleolar proteins, we set the associated observation to *x*_*k *_= 1. For data columns *k *= 5, *k *= 6, *k *= 7 we used the yeast protein complex data sets of Gavin *et al*. [54], Ho *et al*. [55] and Krogan *et al*. [56]. Whenever a yeast protein interacted with a nucleolar protein through shared participation in a complex, we set the associated observation to *x*_*k *_= 1. The resulting data matrix with *K *observations for each yeast protein was used for the prediction of new nucleolar proteins.

### The naïve Bayesian classifier

We use a Bayesian formalism to contrast the hypothesis that a given protein is nucleolar (*H *= *nuc*) with the hypothesis that it is not nucleolar (*H *= nuc¯
 MathType@MTEF@5@5@+=feaafiart1ev1aaatCvAUfeBSjuyZL2yd9gzLbvyNv2Caerbhv2BYDwAHbqedmvETj2BSbqee0evGueE0jxyaibaiKI8=vI8tuQ8FMI8Gi=hEeeu0xXdbba9frFj0=OqFfea0dXdd9vqai=hGuQ8kuc9pgc9s8qqaq=dirpe0xb9q8qiLsFr0=vr0=vr0dc8meaabaqaciGacaGaaeqabaqadeqadaaakeaadaqdaaqaaiaad6gacaWG1bGaam4yaaaaaaa@3613@). According to Bayes rule, the conditional probability that a protein is nucleolar given its associated data x→
 MathType@MTEF@5@5@+=feaafiart1ev1aaatCvAUfeBSjuyZL2yd9gzLbvyNv2Caerbhv2BYDwAHbqedmvETj2BSbqee0evGueE0jxyaibaiKI8=vI8tuQ8FMI8Gi=hEeeu0xXdbba9frFj0=OqFfea0dXdd9vqai=hGuQ8kuc9pgc9s8qqaq=dirpe0xb9q8qiLsFr0=vr0=vr0dc8meaabaqaciGacaGaaeqabaqadeqadaaakeaaceWG4bGbaSaaaaa@343C@ is:

P(nuc|x→)=P(x→|nuc)×P(nuc)P(x→)     (1)
 MathType@MTEF@5@5@+=feaafiart1ev1aaatCvAUfeBSjuyZL2yd9gzLbvyNv2Caerbhv2BYDwAHbqedmvETj2BSbqee0evGueE0jxyaibaiKI8=vI8tuQ8FMI8Gi=hEeeu0xXdbba9frFj0=OqFfea0dXdd9vqai=hGuQ8kuc9pgc9s8qqaq=dirpe0xb9q8qiLsFr0=vr0=vr0dc8meaabaqaciGacaGaaeqabaqadeqadaaakeaacaWGqbGaaiikaiaad6gacaWG1bGaam4yaiaacYhaceWG4bGbaSaacaGGPaGaeyypa0ZaaSaaaeaacaWGqbGaaiikaiqadIhagaWcaiaacYhacaWGUbGaamyDaiaadogacaGGPaGaey41aqRaamiuaiaacIcacaWGUbGaamyDaiaadogacaGGPaaabaGaamiuaiaacIcaceWG4bGbaSaacaGGPaaaaiaaxMaacaWLjaWaaeWaaeaacaaIXaaacaGLOaGaayzkaaaaaa@5046@

where *P*(x→
 MathType@MTEF@5@5@+=feaafiart1ev1aaatCvAUfeBSjuyZL2yd9gzLbvyNv2Caerbhv2BYDwAHbqedmvETj2BSbqee0evGueE0jxyaibaiKI8=vI8tuQ8FMI8Gi=hEeeu0xXdbba9frFj0=OqFfea0dXdd9vqai=hGuQ8kuc9pgc9s8qqaq=dirpe0xb9q8qiLsFr0=vr0=vr0dc8meaabaqaciGacaGaaeqabaqadeqadaaakeaaceWG4bGbaSaaaaa@343C@ | *nuc*) is the likelihood *L*(*nuc*) of the data x→
 MathType@MTEF@5@5@+=feaafiart1ev1aaatCvAUfeBSjuyZL2yd9gzLbvyNv2Caerbhv2BYDwAHbqedmvETj2BSbqee0evGueE0jxyaibaiKI8=vI8tuQ8FMI8Gi=hEeeu0xXdbba9frFj0=OqFfea0dXdd9vqai=hGuQ8kuc9pgc9s8qqaq=dirpe0xb9q8qiLsFr0=vr0=vr0dc8meaabaqaciGacaGaaeqabaqadeqadaaakeaaceWG4bGbaSaaaaa@343C@ under the hypothesis *H *= *nuc*. The conditional probability that a protein is not nucleolar is assigned accordingly.

The posterior odds ratio *O*_*post *_reflects how much more likely it is that a particular protein m is nucleolar than that it is not:

Opost=P(nuc|x→)P(nuc¯|x→)=P(x→|nuc)P(x→|nuc¯)×P(nuc)P(nuc¯)=LR×Oprior     (2)
 MathType@MTEF@5@5@+=feaafiart1ev1aaatCvAUfeBSjuyZL2yd9gzLbvyNv2Caerbhv2BYDwAHbqedmvETj2BSbqee0evGueE0jxyaibaiKI8=vI8tuQ8FMI8Gi=hEeeu0xXdbba9frFj0=OqFfea0dXdd9vqai=hGuQ8kuc9pgc9s8qqaq=dirpe0xb9q8qiLsFr0=vr0=vr0dc8meaabaqaciGacaGaaeqabaqadeqadaaakeaacaWGpbWaaSbaaSqaaiaadchacaWGVbGaam4CaiaadshaaeqaaOGaeyypa0ZaaSaaaeaacaWGqbGaaiikaiaad6gacaWG1bGaam4yaiaacYhaceWG4bGbaSaacaGGPaaabaGaamiuaiaacIcadaqdaaqaaiaad6gacaWG1bGaam4yaaaacaGG8bGabmiEayaalaGaaiykaaaacqGH9aqpdaWcaaqaaiaadcfacaGGOaGabmiEayaalaGaaiiFaiaad6gacaWG1bGaam4yaiaacMcaaeaacaWGqbGaaiikaiqadIhagaWcaiaacYhadaqdaaqaaiaad6gacaWG1bGaam4yaaaacaGGPaaaaiabgEna0oaalaaabaGaamiuaiaacIcacaWGUbGaamyDaiaadogacaGGPaaabaGaamiuaiaacIcadaqdaaqaaiaad6gacaWG1bGaam4yaaaacaGGPaaaaiabg2da9iaadYeacaWGsbGaey41aqRaam4tamaaBaaaleaacaWGWbGaamOCaiaadMgacaWGVbGaamOCaaqabaGccaWLjaGaaCzcamaabmaabaGaaGOmaaGaayjkaiaawMcaaaaa@7102@

The prior odds ratio *O*_*prior *_expresses the prior belief that an unknown protein is nucleolar before seeing its associated data. A lower bound on this prior is estimated from current knowledge about the number of nucleolar proteins (439) and the number of total proteins (6,720) in yeast. We set *O*_*prior *_= 439/(6,720 - 439). The first term in the last equation is the likelihood ratio *LR *= *L*(*nuc*)/*L*(nuc¯
 MathType@MTEF@5@5@+=feaafiart1ev1aaatCvAUfeBSjuyZL2yd9gzLbvyNv2Caerbhv2BYDwAHbqedmvETj2BSbqee0evGueE0jxyaibaiKI8=vI8tuQ8FMI8Gi=hEeeu0xXdbba9frFj0=OqFfea0dXdd9vqai=hGuQ8kuc9pgc9s8qqaq=dirpe0xb9q8qiLsFr0=vr0=vr0dc8meaabaqaciGacaGaaeqabaqadeqadaaakeaadaqdaaqaaiaad6gacaWG1bGaam4yaaaaaaa@3613@) of the data given a pair of hypotheses (here *H *= *nuc *or *H *= nuc¯
 MathType@MTEF@5@5@+=feaafiart1ev1aaatCvAUfeBSjuyZL2yd9gzLbvyNv2Caerbhv2BYDwAHbqedmvETj2BSbqee0evGueE0jxyaibaiKI8=vI8tuQ8FMI8Gi=hEeeu0xXdbba9frFj0=OqFfea0dXdd9vqai=hGuQ8kuc9pgc9s8qqaq=dirpe0xb9q8qiLsFr0=vr0=vr0dc8meaabaqaciGacaGaaeqabaqadeqadaaakeaadaqdaaqaaiaad6gacaWG1bGaam4yaaaaaaa@3613@). The likelihood ratio contains all information on how we should update our prior belief that a particular protein is nucleolar in the light of its associated data. Thus, the posterior odds ratio can be thought of as an updated version of the prior odds ratio after the data have been seen.

How is the likelihood ratio *LR *calculated? In naïve Bayesian classification one 'naïvely' assumes that the observations from different data sources are independent. Then, the likelihood ratio of a complete set of observed data points is just the product of the likelihood ratios for individual observations:

LR=∏k=1KLRk=∏k=1KLk(nuc)Lk(nuc¯)     (3)
 MathType@MTEF@5@5@+=feaafiart1ev1aaatCvAUfeBSjuyZL2yd9gzLbvyNv2Caerbhv2BYDwAHbqedmvETj2BSbqee0evGueE0jxyaibaiKI8=vI8tuQ8FMI8Gi=hEeeu0xXdbba9frFj0=OqFfea0dXdd9vqai=hGuQ8kuc9pgc9s8qqaq=dirpe0xb9q8qiLsFr0=vr0=vr0dc8meaabaqaciGacaGaaeqabaqadeqadaaakeaacaWGmbGaamOuaiabg2da9maarahabaGaamitaiaadkfadaWgaaWcbaGaam4AaaqabaaabaGaam4Aaiabg2da9iaaigdaaeaacaWGlbaaniabg+GivdGccqGH9aqpdaqeWbqaamaalaaabaGaamitamaaBaaaleaacaWGRbaabeaakiaacIcacaWGUbGaamyDaiaadogacaGGPaaabaGaamitamaaBaaaleaacaWGRbaabeaakiaacIcadaqdaaqaaiaad6gacaWG1bGaam4yaaaacaGGPaaaaaWcbaGaam4Aaiabg2da9iaaigdaaeaacaWGlbaaniabg+GivdGccaWLjaGaaCzcamaabmaabaGaaG4maaGaayjkaiaawMcaaaaa@5507@

The individual likelihoods *L*_*k*_(*H*), *H *∈ {*nuc*, nuc¯
 MathType@MTEF@5@5@+=feaafiart1ev1aaatCvAUfeBSjuyZL2yd9gzLbvyNv2Caerbhv2BYDwAHbqedmvETj2BSbqee0evGueE0jxyaibaiKI8=vI8tuQ8FMI8Gi=hEeeu0xXdbba9frFj0=OqFfea0dXdd9vqai=hGuQ8kuc9pgc9s8qqaq=dirpe0xb9q8qiLsFr0=vr0=vr0dc8meaabaqaciGacaGaaeqabaqadeqadaaakeaadaqdaaqaaiaad6gacaWG1bGaam4yaaaaaaa@3613@} can easily be calculated from the positive and negative training data, that is, sets of proteins that are nucleolar and that are not nucleolar and their associated data. Individual data points *x*_*k *_are binary. Let all *n*(*H*), *H *∈ {*nuc*, nuc¯
 MathType@MTEF@5@5@+=feaafiart1ev1aaatCvAUfeBSjuyZL2yd9gzLbvyNv2Caerbhv2BYDwAHbqedmvETj2BSbqee0evGueE0jxyaibaiKI8=vI8tuQ8FMI8Gi=hEeeu0xXdbba9frFj0=OqFfea0dXdd9vqai=hGuQ8kuc9pgc9s8qqaq=dirpe0xb9q8qiLsFr0=vr0=vr0dc8meaabaqaciGacaGaaeqabaqadeqadaaakeaadaqdaaqaaiaad6gacaWG1bGaam4yaaaaaaa@3613@} be the number of proteins in the training data that fulfill hypothesis H. Let all *n*(*x*_*k*_, *H*), *H *∈ {*nuc*, nuc¯
 MathType@MTEF@5@5@+=feaafiart1ev1aaatCvAUfeBSjuyZL2yd9gzLbvyNv2Caerbhv2BYDwAHbqedmvETj2BSbqee0evGueE0jxyaibaiKI8=vI8tuQ8FMI8Gi=hEeeu0xXdbba9frFj0=OqFfea0dXdd9vqai=hGuQ8kuc9pgc9s8qqaq=dirpe0xb9q8qiLsFr0=vr0=vr0dc8meaabaqaciGacaGaaeqabaqadeqadaaakeaadaqdaaqaaiaad6gacaWG1bGaam4yaaaaaaa@3613@} be the number of proteins in the training data that fulfill hypothesis H and have associated data *x*_*k *_(either 0 or 1). Thus, for a new protein the likelihoods *L*_*k*_(*H*), *H *∈ {*nuc*, nuc¯
 MathType@MTEF@5@5@+=feaafiart1ev1aaatCvAUfeBSjuyZL2yd9gzLbvyNv2Caerbhv2BYDwAHbqedmvETj2BSbqee0evGueE0jxyaibaiKI8=vI8tuQ8FMI8Gi=hEeeu0xXdbba9frFj0=OqFfea0dXdd9vqai=hGuQ8kuc9pgc9s8qqaq=dirpe0xb9q8qiLsFr0=vr0=vr0dc8meaabaqaciGacaGaaeqabaqadeqadaaakeaadaqdaaqaaiaad6gacaWG1bGaam4yaaaaaaa@3613@} are calculated as follows:

Lk(nuc)=P(xk|nuc)=n(xk,nuc)n(nuc)     (4)
 MathType@MTEF@5@5@+=feaafiart1ev1aaatCvAUfeBSjuyZL2yd9gzLbvyNv2Caerbhv2BYDwAHbqedmvETj2BSbqee0evGueE0jxyaibaiKI8=vI8tuQ8FMI8Gi=hEeeu0xXdbba9frFj0=OqFfea0dXdd9vqai=hGuQ8kuc9pgc9s8qqaq=dirpe0xb9q8qiLsFr0=vr0=vr0dc8meaabaqaciGacaGaaeqabaqadeqadaaakeaacaWGmbWaaSbaaSqaaiaadUgaaeqaaOGaaiikaiaad6gacaWG1bGaam4yaiaacMcacqGH9aqpcaWGqbGaaiikaiaadIhadaWgaaWcbaGaam4AaaqabaGccaGG8bGaamOBaiaadwhacaWGJbGaaiykaiabg2da9maalaaabaGaamOBaiaacIcacaWG4bWaaSbaaSqaaiaadUgaaeqaaOGaaiilaiaad6gacaWG1bGaam4yaiaacMcaaeaacaWGUbGaaiikaiaad6gacaWG1bGaam4yaiaacMcaaaGaaCzcaiaaxMaadaqadaqaaiaaisdaaiaawIcacaGLPaaaaaa@5434@

Lk(nuc¯)=P(xk|nuc¯)=n(xk,nuc¯)n(nuc¯)     (5)
 MathType@MTEF@5@5@+=feaafiart1ev1aaatCvAUfeBSjuyZL2yd9gzLbvyNv2Caerbhv2BYDwAHbqedmvETj2BSbqee0evGueE0jxyaibaiKI8=vI8tuQ8FMI8Gi=hEeeu0xXdbba9frFj0=OqFfea0dXdd9vqai=hGuQ8kuc9pgc9s8qqaq=dirpe0xb9q8qiLsFr0=vr0=vr0dc8meaabaqaciGacaGaaeqabaqadeqadaaakeaacaWGmbWaaSbaaSqaaiaadUgaaeqaaOGaaiikamaanaaabaGaamOBaiaadwhacaWGJbaaaiaacMcacqGH9aqpcaWGqbGaaiikaiaadIhadaWgaaWcbaGaam4AaaqabaGccaGG8bWaa0aaaeaacaWGUbGaamyDaiaadogaaaGaaiykaiabg2da9maalaaabaGaamOBaiaacIcacaWG4bWaaSbaaSqaaiaadUgaaeqaaOGaaiilamaanaaabaGaamOBaiaadwhacaWGJbaaaiaacMcaaeaacaWGUbGaaiikamaanaaabaGaamOBaiaadwhacaWGJbaaaiaacMcaaaGaaCzcaiaaxMaadaqadaqaaiaaiwdaaiaawIcacaGLPaaaaaa@5479@

### Training the classifier and estimation of classification performance

To train our classifier for the prediction of new nucleolar proteins we needed positive and negative training data, that is, sets of known nucleolar and non-nucleolar proteins. We retrieved overlapping lists of 219 known nucleolar proteins, 239 proteins acting in ribosome biosynthesis, and 159 proteins associated with cytosolic ribosomes from the SGD. This resulted in a non-redundant set of 439 nucleolar proteins, which we used as positive training cases.

The acquisition of negative training cases was not as straightforward, because we suspect that, among the remaining 6,720 - 439 = 6,281 yeast proteins, a considerable number are nucleolar ones. We consciously decided not to chose a biologically motivated approach to acquire negative training examples to avoid introducing an unknown biological bias into the negative training set (for example, by taking only extracellular or organelle proteins as negative training data). Instead, we obtained 1,000 random samples of 439 proteins (the same size as the positive set) from all but the 439 nucleolar proteins.

Each sample of negative training cases was combined with the unique positive training set to yield a complete training data set. For each of these 1,000 training data sets we performed 10-fold cross-validation. We applied a range of thresholds to determine the sensitivity (*SE *= *TP*/(*TP *+ *FN*)) and specificity (*SP *= *TN*/(*TN *+ *FP*)) and the ROC curve for each of the 1,000 cross-validation runs. We determined the average AUC from 1,000 cross-validation runs to judge the quality of our classifier.

### Prediction of novel nucleolus or ribosome-associated proteins

After encouraging cross-validation results, we tried to predict new nucleolar proteins from the set of 6,281 proteins not previously assigned as nucleolar using a similar strategy. We randomly sampled 1,000 sets of 439 negative training cases from the 6,281 proteins and combined each with the 439 positive training cases, thus yielding 1,000 training data sets. We built 1,000 classifiers from these training data sets. With each classifier we made predictions for all proteins not used for training. For a single prediction we used a threshold of *log*(*O*_*post*_) = 0.4. Application of this threshold led to a sensitivity of 50.5% and a specificity of 98.6% during cross-validation. In total, we obtained approximately 900 predictions for each protein (less than 1,000 because we only considered predictions in which the protein was not used for training). The actual classifier decision as to whether a single protein is nucleolar or not was a majority vote based on all approximately 900 predictions.

Based on the prediction results (see Results and discussion; Figure 1), we estimate that our set of 6,281 non-NRCA proteins - for which we assumed that the majority of cases are not NRCA proteins - does probably contain approximately 124 positives (1.97%). Thus, in retrospect, we estimate that, on average, 9 of the presumed non-NRCA proteins are actually positive when we sample 439 proteins at random to compile a negative training set. This probably led to a slight reduction in sensitivity and specificity of the classifier. However, as we can not compile a better set of negative training cases without introducing a systematic bias, we argue that repeated random sampling of negative training cases is an adequate procedure for this classification problem.

### Phylogenetic profiling of yeast nucleolar proteins

We obtained sequences from 84 complete proteomes from the ftp server of the European Bioinformatics Institute or the genome download sites at the Wellcome Trust Sanger Institute, The Institute for Genomics Research (TIGR), and the Marine Biological Laboratory. Protein sequences of *S. cerevisiae *were retrieved from the Munich information center for protein sequences [57-60]. The proteomes of the following organisms were used as target proteomes to derive phylogenetic profiles for yeast proteins that served us as reference proteins. Eukaryota: ashgos, *Ashbya gossypii*; klulac, *Kluyveromyces lactis*; schpom, *Schizosaccharomyces pombe*; aspnid, *Aspergillus nidulans*; homsap, *Homo sapiens*; ratnov, *Rattus norvegicus*; musmus, *Mus musculus*; galgal, *Gallus gallus*; danrer, *Danio rerio*; dromel, *Drosophila melanogaster*; caeele, *Caenorhabditis elegans*; enccun, *Encephalitozoon cuniculi*; dicdis, *Dictyostelium discoideum*; chlrei, *Chlamydomonas reinhardtii*; guithe, *Guillardia theta*; cyamer, *Cyanidioschyzon merolae*; aratha, *Arabidopsis thaliana*; plafal, *Plasmodium falciparum*; leimaj, *Leishmania major*; gialam, *Giadia lamblia*. Archaea: nanequ, *Nanoarchaeum equitans*; aerper, *Aeropyrum pernix*; pyraer, *Pyrobaculum aerophilum*; sulsol, *Sulfolobus solfataricus*; sultok, *Sulfolobus tokodaii*; arcful, *Archaeoglobus fulgidus*; halnrc, *Halobacterium *sp.; halmar, *Haloarcula marismortui*; metthe, *Methanobacterium thermoautotrophicum*; metjan, *Methanococcus jannaschii*; metmar, *Methanococcus maripaludis*; metkan, *Methanopyrus kandleri*; metace, *Methanosarcina acetivorans*; metmaz, *Methanosarcina mazei*; pyraby, *Pyrococcus abyssi*; pyrfur, *Pyrococcus furiosus*; pyrhor, *Pyrococcus horikoshii*; theaci, *Thermoplasma acidophilum*; thevol, *Thermoplasma volcanium*; pictor, *Picrophilus torridus*. Bacteria: anaspe, *Anabaena *sp.; synelo, *Synechococcus elongates*; synspe, *Synechocystis *sp.; rhilot, *Rhizobium loti*; riccon, *Rickettsia conorii*; ricpro, *Rickettsia prowazekii*; agrtum, *Agrobacterium tumefaciens*; brajap, *Bradyrhizobium japonicum*; wolpip, *Wolbachia pipientis*; niteur, *Nitrosomonas europaea*; ralsol, *Ralstonia solanacearum*; burmal, *Burkholderia mallei*; neimea, *Neisseria meningitis*; pseaer, *Pseudomonas aeruginosa*; esccol, *Escherichia coli*; haeinf, *Haemophilus influenzae*; saltyp, *Salmonella typhimurium*; sheone, *Shewanella oneidensis*; vibcol, *Vibrio cholerae*; wigglo, *Wigglesworthia glossinidia brevipalpis*; xancam, *Xanthomonas campestris*; xylfas, *Xylella fastidiosa*; yerpes, *Yersinia pestis*; camjej, *Campylobacter jejuni*; helpyl, *Helicobacter pylori*; corglu, *Corynebacterium glutamicum*; strcoe, *Streptomyces coelicolor*; trowhi, *Tropheryma whipplei*; symthe, *Symbiobacterium thermophilum*; trepal, *Treponema pallidum*; borbur, *Borrelia burgdorferi*; lepint, *Leptospira interrogans*; backer, *Bacillus cereus*; cloace, *Clostridium acetobutylicum*; lismon, *Listeria monocytogenes*; mycpne, *Mycoplasma pneumoniae*; oceihe, *Oceanobacillus iheyensis*; staepi, *Staphylococcus epidermidis*; strpyo, *Streptococcus pyogenes*; themar, *Thermotoga maritime*; bacthe, *Bacteroides thetaiotaomicron*; chlmur, *Chlamydia muridarum*; aquael, *Aquifex aeolicus*; deirad, *Deinococcus radiodurans*.

We applied the 'best reciprocal hit' (BRH) method to find orthologous protein pairs between the reference proteome and a target proteome. The BRH method is an approximative method for ortholog identification that has been applied by many other groups before to identify orthologs for pairs of organisms with considerable accuracy (see, for example, [61-65]. The BRH method performs worse than more sophisticated phylogeny-based techniques (like reconciliation of phylogenetic and species tree), but, because of its simplicity, it is especially suited for phylogenetic profiling of proteomes of dozens of organisms. More advanced schemes based on pairwise sequence matching (for example, INPARANOID) are also able to find so called 'in-paralogs' (paralogs in one organism that have to be called orthologous to a protein in a second organism with equal right, because they emerged from a duplication after the evolutionary split of these organisms). For ortholog phylogenetic profiling, detection of such paralogs is not so important because the profile scores come from the best hit anyway. Compared to clustering approaches for ortholog identification (COG, orthoMCL), the BRH method relies only on pairwise comparisons of a reference proteome with target genomes and, therefore, more closely adheres to the original definition of orthology, which is always defined between two species. For a more detailed discussion of the BRH method for phylogenetic profiling we refer to our recent study [66].

In our implementation of BRH-based phylogenetic profiling we used yeast proteins as queries to carry out BLASTP searches [67] against each of the 84 proteomes. The best hits for each yeast protein in the individual proteomes were recorded. Then, we used those protein sequences that were identified as best hits as queries in reciprocal BLAST searches of the complete yeast proteome. For BLAST searches we used default parameters (BLOSUM62 matrix, SEG filter on, gap open penalty: 11, gap extension penalty: 1) and an expectation (E) value threshold of *E *< 0.1. Only reciprocal best hits were considered for the construction and visualization of phylogenetic profiles. For visualization, the E values of prokaryote-versus-yeast-proteome searches were color-coded in a yeast-protein-versus-prokaryotic-species matrix, our phylogenetic profile, using white and various levels of gray.

For clustering of phylogenetic profiles, we obtained binary (presence-absence/1 or 0) phylogenetic profiles for all nucleolar proteins of yeast identified here. These profiles were subjected to hierarchical clustering using the centroid method and city-block distances using the software CLUSTER 3.0 [68]. The result helped us to identify sets of nucleolar proteins that stem from archaea, bacteria, or emerged late in eukaryotes. We visualized the results using the Java Treeview software [69] or our own phylogenetic profile viewer [70].

### Expression analysis of nucleolar protein components

We performed an analysis of nucleolar expression patterns across 300 experimental conditions of the ROSETTA yeast expression compendium [47]. We aimed to compare co-expression of genes within or between several functionally or evolutionary related groups of genes. Therefore, we determined Pearson correlation coefficients for pairs of genes within or between groups that were calculated from their paired vectors of logarithmic expression ratios across different experimental conditions. We investigated groups that were either identified during the preceding analysis (proteins of the nucleolus, archaeal proteins of the nucleolus, non-archaeal proteins of the nucleolus) or obtained from external resources (the cytosolic ribosome components as recorded by SGD and Gene Ontology [71], 90S processosome proteins listed by Fromont-Racine *et al*. [3]). Histograms of correlation coefficients were determined for each group or group cross-comparison to visualize the degree of co-regulation. Additionally, the expression of genes encoding the cytosolic ribosome and the 90S processosome were investigated using the CLUSTER software (version 3.0). We performed a hierarchical clustering of the original logarithmic expression ratios of these genes using the centroid method and the un-centered correlation option. We visualized the results using the Java Treeview software [69].

## Additional data files

The following additional data are available with the online version of this manuscript. Additional data file 1 contains information about known nucleolar proteins and their results during classifier cross-validation. Additional data file 2 contains all classification results for proteins not predicted as nucleolar.

## Supplementary Material

Additional data file 1 The first line contains abbreviations describing the column content: YORF, yeast open reading frame ID; Gene, gene symbol of yeast gene; Hs, orthology to human nucleolar protein; At, orthology to mouse ear cress nucleolar protein; It, link to nucleolar protein via Y2H interaction in Ito dataset; Ue, link to nucleolar protein via Y2H interaction in Uetz dataset; Ga, link to nucleolar protein via participation in a complex in Gavin data set; Ho, link to nucleolar protein via participation in a complex in Ho data set; Kr, link to nucleolar protein via participation in a complex in Krogan data set; log(O), average posterior odds ratio *O*_*post *_from all cross-validation runs (note that the proteins served as positive training cases); Pred., prediction result (nucleolar or not) according to a majority vote based on all cross-validation runs; Desc., concise description of protein function. Click here for file

Additional file 2 The first line contains abbreviations describing the column content. Gene, gene symbol of yeast gene; ORF, yeast open reading frame ID; Hs, orthology to human nucleolar protein; At, orthology to mouse ear cress nucleolar protein; It, link to nucleolar protein via Y2H interaction in Ito dataset; Ue, link to nucleolar protein via Y2H interaction in Uetz dataset; Ga, link to nucleolar protein via participation in a complex in Gavin data set; Ho, link to nucleolar protein via participation in a complex in Ho data set; Kr, link to nucleolar protein via participation in a complex in Krogan data set; log(O), average posterior odds ratio from all prediction runs in which the protein was not used for training; Desc., concise description of protein function. The listed proteins were all predicted to be not nucleolar based on a threshold of *O*_*post *_< 0.4. Click here for file

## Figures and Tables

**Table 1:** Classification results and annotation for 62 novel predicted nucleolar/ribosome-associated proteins

Gene	ORF	Hs	At	Ue	It	Kr	Ga	Ho	log(O)	Description
SUA7	YPR086W	1	0	1	0	1	0	o	0.665	TFIIB subunit (transcription initiation factor) factor E
HTA1	YDR225W	1	0	1	0	0	1	0	0.612	Histone H2A
HSC82	YMR186W	1	1	0	0	1	0	0	0.697	Heat shock protein
TIF1	YKR059W	1	1	0	0	1	0	0	0.699	Translation initiation factor 4A
PRP4	YPR178W	1	0	0	0	1	0	1	0.703	U4/U6 snRNP 52 kDa protein
KAR2	YJL034W	1	1	0	0	0	0	0	0.684	Component of ER translocon
HTA2	YBL003C	1	0	0	0	1	1	0	0.724	Histone H2A.2
AAC3	YBR085W	1	1	0	0	0	1	0	0.686	Mitochondrial ADP/ATP carrier - member of the mitochondrial carrier (MCF) family
RFC2	YJR068W	1	1	0	0	0	0	0	0.686	DNA replication factor C 41 kDa subunit
TEF1	YPR080W	1	1	0	0	0	0	0	0.686	Translation elongation factor eEF1 alpha-A chain cytosolic
SMX2	YFL017W-A	1	1	0	0	1	0	0	0.696	snRNP G protein (the homologue of the human Sm-G)
BCP1	YDR361C	1	1	0	0	0	0	0	0.686	Similarity to hypothetical protein *S. pombe*
LEA1	YPL213W	1	1	0	0	1	0	0	0.704	U2 A snRNP protein
HSP82	YPL240C	1	1	0	0	0	0	0	0.686	Heat shock protein
SMD3	YLR147C	1	1	0	0	1	0	0	0.699	Spliceosomal snRNA-associated Sm core protein required for pre-mRNA splicing
TIF2	YJL138C	1	1	0	0	0	1	0	0.686	Translation initiation factor eIF4A
None	YBR025C	1	0	1	0	0	1	0	0.610	Strong similarity to Ylf1p
SPT16	YGL207W	1	1	0	0	1	1	0	0.705	General chromatin factor
SUI2	YJR007W	1	0	0	0	1	1	0	0.720	Translation initiation factor eIF2 alpha chain
HSH49	YOR319W	0	1	0	1	0	1	0	0.702	Essential yeast splicing factor
DED1	YOR204W	0	1	0	0	0	0	1	0.716	ATP-dependent RNA helicase
HTB1	YDR224C	1	1	1	0	0	1	0	0.709	Histone H2B
HRR25*	YPL204W	1	0	0	0	1	1	0	0.718	Casein kinase I Ser/Thr/Tyr protein kinase
SSA2	YLL024C	1	1	0	0	0	0	0	0.686	Heat shock protein of HSP70 family cytosolic
SRP1	YNL189W	0	1	1	0	1	1	1	0.696	Karyopherin-alpha or importin
SUB2	YDL084W	1	1	0	0	0	0	0	0.686	Probably involved in pre-mRNA splicing
CKA1	YIL035C	1	0	0	0	1	1	1	0.698	Casein kinase II catalytic alpha chain
PRP43*	YGL120C	1	1	1	0	1	1	0	0.695	Involved in spliceosome disassembly
SUI3	YPL237W	1	0	0	0	1	1	0	0.721	Translation initiation factor eIF2 beta subunit
DST1	YGL043W	1	0	0	0	0	0	1	0.692	TFIIS (transcription elongation factor)
PRP8	YHR165C	1	0	0	0	1	1	0	0.721	U5 snRNP protein pre-mRNA splicing factor
PRP9	YDL030W	1	0	1	0	1	0	0	0.667	Pre-mRNA splicing factor (snRNA-associated protein)
SUP45	YBR143C	1	0	1	0	1	1	0	0.704	Translational release factor
ASC1	YMR116C	1	1	0	0	1	0	0	0.698	40S small subunit ribosomal protein
DBP2*	YNL112W	1	0	0	0	1	1	0	0.719	ATP-dependent RNA helicase of DEAD box family
CKB2	YOR039W	1	0	0	1	1	1	0	0.710	Casein kinase II beta chain
YRA1	YDR381W	1	0	0	0	1	1	0	0.720	RNA annealing protein
GCD11	YER025W	1	0	1	0	0	1	0	0.609	Translation initiation factor eIF2 gamma chain
TFG2	YGR005C	1	0	0	0	1	1	1	0.695	TFIIF subunit (transcription initiation factor) 54 kDa
TOP1*	YOL006C	1	0	1	1	1	0	0	0.693	DNA topoisomerase I
BRR2	YER172C	1	1	0	0	0	0	1	0.708	RNA helicase-related protein
RVB1	YDR190C	1	1	1	0	0	1	0	0.709	RUVB-like protein
MLP1	YKR095W	1	1	0	0	0	0	0	0.686	Myosin-like protein related to Uso1p
HTZ1	YOL012C	1	1	0	0	0	0	0	0.685	Evolutionarily conserved member of the histone H2A F/Z family of histone variants
ATP2	YJR121W	1	1	0	0	0	0	0	0.685	F1F0-ATPase complex F1 beta subunit
SMD2	YLR275W	1	1	0	1	1	0	0	0.688	U1 snRNP protein of the Sm class
PRP3	YDR473C	1	0	0	0	1	0	1	0.704	Essential splicing factor
EFT1	YOR133W	1	1	0	0	0	0	0	0.682	Translation elongation factor eEF2
HTB2	YBL002W	1	1	0	0	0	1	0	0.690	Histone H2B.2
TEF4	YKL081W	1	0	0	0	1	1	0	0.718	Translation elongation factor eEF1 gamma chain
HHF2	YNL030W	1	1	0	0	1	0	0	0.695	Histone H4
Predictions based solely on protein interactions										
RPO21	YDL140C	0	0	0	0	1	1	1	0.728	DNA-directed RNA polymerase II 215 kDa subunit
DHH1	YDL160C	0	0	1	1	1	1	0	0.714	Putative RNA helicase of the DEAD box family
CFT1	YDR301W	0	0	0	0	1	1	1	0.731	Pre-mRNA 3-end processing factor CF II
KAP95	YLR347C	0	0	1	0	1	1	1	0.689	Karyopherin-beta
SPT5	YML010W	0	0	0	0	1	1	1	0.732	Transcription elongation protein
TAF14	YPL129W	0	0	0	0	1	1	1	0.733	TFIIF subunit (transcription initiation factor) 30 kDa
RPB3	YIL021W	0	0	0	0	1	1	1	0.728	DNA-directed RNA-polymerase II 45 kDa
RPO31	YOR116C	0	0	0	0	1	1	1	0.729	DNA-directed RNA polymerase III 160 kDa subunit
TIF4631	YGR162W	0	0	0	0	1	1	1	0.734	mRNA cap-binding protein (eIF4F) 150K subunit
PRP24	YMR268C	0	0	0	0	1	1	1	0.734	Pre-mRNA splicing factor
RET1	YOR207C	0	0	0	0	1	1	1	0.731	DNA-directed RNA polymerase III 130 kDa subunit

The data used for classification and the detailed prediction results are listed for all 62 proteins that passed our threshold of *O*_*post *_> 0.4. These proteins had not been annotated as associated with nucleolar or ribosomal components before, but were classified as such in our analysis. A literature survey for the predicted proteins revealed that for four proteins a role in the nucleolus and ribosome biogenesis had already been established (see Note added in proof). The lower part of the table lists 11 proteins that were predicted as NRCA proteins solely on the basis of shared participation in complexes or interactions. For these proteins, we do not necessarily predict a nucleolar localization, but direct interaction with nucleolar/ribosomal components at least under one specific cellular condition at an unspecified locus within the cell. *Four proteins for which recent articles have confirmed a role in ribosome biogenesis or the nucleolus. The results are supplemented by a concise annotation for each protein from the Comprehensive Yeast Genome Database (CYGD) [72]. The header line contains abbreviations describing the column content: Gene, gene symbol of yeast gene; ORF, yeast open reading frame ID; Hs, orthology to human nucleolar protein; At, orthology to mouse-ear cress nucleolar protein; It, link to nucleolar protein via Y2H interaction in Ito dataset; Ue, link to nucleolar protein via Y2H interaction in Uetz dataset; Ga, link to nucleolar protein via participation in a complex in Gavin data set; Ho, link to nucleolar protein via participation in a complex in Ho data set; Kr, link to nucleolar protein via participation in a complex in Krogan data set; log(O), average posterior odds ratio from all prediction runs in which the protein was not used for training; Description, concise description of protein function.

**Table 2:** Summary of effective prediction rules obtained by Bayesian classification

Hs	At	Ue	It	Kr	Ga	Ho	Prediction: associated with nucleolar or ribosomal component?
0	0	0	0	0	0	0	No
0	0	0	0	0	0	1	No
0	0	0	0	0	1	0	No
0	0	0	0	0	1	1	No
0	0	0	0	1	0	0	No
0	0	0	0	1	0	1	No
0	0	0	0	1	1	0	No
0	0	0	1	0	0	0	No
0	0	0	1	0	1	0	No
0	0	0	1	1	0	0	No
0	0	0	1	1	1	0	No
0	0	1	0	0	0	0	No
0	0	1	0	0	0	1	No
0	0	1	0	0	1	0	No
0	0	1	0	1	0	0	No
0	0	1	0	1	1	0	No
0	0	1	1	0	0	0	No
0	0	1	1	0	1	0	No
0	1	0	0	0	0	0	No
0	1	0	0	0	1	0	No
0	1	0	0	1	0	0	No
1	0	0	0	0	0	0	No
1	0	0	0	0	1	0	No
1	0	0	0	1	0	0	No
1	0	0	1	0	0	0	No
1	0	1	0	0	0	0	No
0	0	0	0	1	1	1	Yes
0	0	1	0	1	1	1	Yes
0	0	1	1	1	1	0	Yes
0	1	0	0	0	0	1	Yes
0	1	0	1	0	1	0	Yes
0	1	1	0	1	1	1	Yes
1	0	0	0	0	0	1	Yes
1	0	0	0	1	0	1	Yes
1	0	0	0	1	1	0	Yes
1	0	0	0	1	1	1	Yes
1	0	0	1	1	1	0	Yes
1	0	1	0	0	1	0	Yes
1	0	1	0	1	0	0	Yes
1	0	1	0	1	1	0	Yes
1	0	1	1	1	0	0	Yes
1	1	0	0	0	0	0	Yes
1	1	0	0	0	0	1	Yes
1	1	0	0	0	1	0	Yes
1	1	0	0	1	0	0	Yes
1	1	0	0	1	1	0	Yes
1	1	0	1	1	0	0	Yes
1	1	1	0	0	1	0	Yes
1	1	1	0	1	1	0	Yes

Our Bayesian classification approach assigns a distinct prediction to each possible binary pattern that could be associated with a protein. With the seven data sources used here, only a limited number of 128 different combinations of binary evidences is possible. Here, all binary patterns that occur in our data set are enumerated. They are supplemented by the prediction result to illustrate which input data generates which prediction. Note that neither a single protein interaction nor a single occurrence in nucleoli of model organisms is sufficient for a positive prediction, and that evidence from three protein interaction experiments is necessary for a positive prediction in the absence of evidence based on orthologs in nucleolar preparations of model organisms. Column headers denote the data source: Hs, orthology to human nucleolar protein; At, orthology to mouse-ear cress nucleolar protein; Ue, link to nucleolar protein via Y2H interaction in Uetz dataset; It, link to nucleolar protein via Y2H interaction in Ito dataset; Kr, link to nucleolar protein via participation in a complex in Krogan data set; Ga, link to nucleolar protein via participation in a complex in Gavin data set; Ho, link to nucleolar protein via participation in a complex in Ho data set.
